# Nephrocalcinosis (Enamel Renal Syndrome) Caused by Autosomal Recessive ***FAM20A*** Mutations

**DOI:** 10.1159/000349989

**Published:** 2013-02-23

**Authors:** Graciana Jaureguiberry, Muriel De la Dure-Molla, David Parry, Mickael Quentric, Nina Himmerkus, Toshiyasu Koike, James Poulter, Enriko Klootwijk, Steven L. Robinette, Alexander J. Howie, Vaksha Patel, Marie-Lucile Figueres, Horia C. Stanescu, Naomi Issler, Jeremy K. Nicholson, Detlef Bockenhauer, Christopher Laing, Stephen B. Walsh, David A. McCredie, Sue Povey, Audrey Asselin, Arnaud Picard, Aurore Coulomb, Alan J. Medlar, Isabelle Bailleul-Forestier, Alain Verloes, Cedric Le Caignec, Gwenaelle Roussey, Julien Guiol, Bertrand Isidor, Clare Logan, Roger Shore, Colin Johnson, Christopher Inglehearn, Suhaila Al-Bahlani, Matthieu Schmittbuhl, François Clauss, Mathilde Huckert, Virginie Laugel, Emmanuelle Ginglinger, Sandra Pajarola, Giuseppina Spartà, Deborah Bartholdi, Anita Rauch, Marie-Claude Addor, Paulo M. Yamaguti, Heloisa P. Safatle, Ana Carolina Acevedo, Hercílio Martelli-Júnior, Pedro E. dos Santos Netos, Ricardo D. Coletta, Sandra Gruessel, Carolin Sandmann, Denise Ruehmann, Craig B. Langman, Steven J. Scheinman, Didem Ozdemir-Ozenen, Thomas C. Hart, P. Suzanne Hart, Ute Neugebauer, Eberhard Schlatter, Pascal Houillier, William A. Gahl, Miikka Vikkula, Agnès Bloch-Zupan, Markus Bleich, Hiroshi Kitagawa, Robert J. Unwin, Alan Mighell, Ariane Berdal, Robert Kleta

**Affiliations:** ^1^Centre for Nephrology, University College London, London, UK; ^2^Rothschild Dental Hospital Service, Paris, France; ^3^Molecular Medicine, University of Leeds, Leeds, UK; ^4^de Duve Institute, Université catholique de Louvain, Brussels, Belgium; ^5^Physiology, University of Kiel, Kiel, Germany; ^6^Biochemistry, Kobe Pharmaceutical University, Kobe, Japan; ^7^Biomolecular Medicine, Imperial College London, London, UK; ^8^Cordeliers Research Center, Paris-Descartes University, Paris, France; ^9^Royal Children's Hospital, Melbourne, Vic., Australia; ^10^Department of Genetics, Evolution and Environment, UCL, London, UK; ^11^INSERM, UMRS 872, University Paris-Diderot, France; ^12^Pathology service, Armand Trousseau Hospital, Paris, France; ^13^Toulouse Hospital, Sabatier University, Toulouse, Nantes, France; ^14^Department of Genetics, APHP – Robert Debré University Hospital, Paris, and Services de, Nantes, France; ^15^Génétique, CHU de Nantes, Nantes, France; ^16^Pédiatrie, CHU de Nantes, Nantes, France; ^17^Stomatologie, CHU de Nantes, Nantes, France; ^18^Leeds Dental Institute, University of Leeds, Leeds, UK; ^19^Al-Nahda Hospital, Muscat, Sultanate of Oman; ^20^University of Strasbourg, Strasbourg, Hôpital Emile Muller, Mulhouse, France; ^21^IGBMC, INSERM, U964, Illkirch, Hôpital Emile Muller, Mulhouse, France; ^22^Service de Génétique, Hôpital Emile Muller, Mulhouse, France; ^23^Medical Genetics, University of Zurich, Lausanne, Switzerland; ^24^Nephrology Unit, University Children's Hospital, Zurich, Lausanne, Switzerland; ^25^Service de Génétique Médicale, Lausanne, Switzerland; ^26^Health Sciences School, University of Brasilia, Brasilia, São Paulo, Brazil; ^27^Department of Medical Genetics, University of Brasilia, Brasilia, São Paulo, Brazil; ^28^State University of Montes Claros, Minas Gerais, São Paulo, Brazil; ^29^Dental School, State University of Campinas, São Paulo, Brazil; ^30^Pediatric Nephrology, Northwestern University, Chicago, Ill., Scranton, Pa., USA; ^31^The Commonwealth Medical College, Scranton, Pa., USA; ^32^Pedodontics, Yeditepe University, Istanbul, Turkey; ^33^Periodontics, University of Illinois at Chicago, Chicago, Ill., Md., USA; ^34^NHGRI, NIH, Bethesda, Md., USA; ^35^Internal Medicine D, University of Muenster, Muenster, Germany

**Keywords:** Nephrolithiasis, Urolithiasis, Amelogenesis imperfecta, FAM20B, FAM20C

## Abstract

**Background/Aims:**

Calcium homeostasis requires regulated cellular and interstitial systems interacting to modulate the activity and movement of this ion. Disruption of these systems in the kidney results in nephrocalcinosis and nephrolithiasis, important medical problems whose pathogenesis is incompletely understood.

**Methods:**

We investigated 25 patients from 16 families with unexplained nephrocalcinosis and characteristic dental defects (amelogenesis imperfecta, gingival hyperplasia, impaired tooth eruption). To identify the causative gene, we performed genome-wide linkage analysis, exome capture, next-generation sequencing, and Sanger sequencing.

**Results:**

All patients had bi-allelic *FAM20A* mutations segregating with the disease; 20 different mutations were identified.

**Conclusions:**

This au-tosomal recessive disorder, also known as enamel renal syndrome, of *FAM20A* causes nephrocalcinosis and amelogenesis imperfecta. We speculate that all individuals with biallelic *FAM20A* mutations will eventually show nephrocalcinosis.

## Introduction

Nephrocalcinosis (NC), diagnosed by radiographs, CT, or increased echogenicity on ultrasound, represents an important renal complication because it can accompany progressive deterioration of glomerular function or nephrolithiasis [1]. In some cases, NC provides a clue to an underlying genetic disorder such as hyperoxaluria or distal renal tubular acidosis with or without deafness [2,3]. In other cases, NC is a side effect of chronic treatment with various drugs, including loop diuretics and vitamin D. In general, the pathogenesis of NC has not been adequately elucidated, although hypercalciuria appears to be a common finding.

Often, rare genetic diseases reveal previously unrecognized mechanisms of action regarding physiology, cell biology, and metabolism. Here, we present genetic studies into a rare human disease of NC combined with amelogenesis imperfecta (AI), a disorder of abnormal enamel formation and impaired tooth eruption.

## Methods

Patients and families were identified in our rare disease renal tubular or dental/craniofacial reference centers and gave informed consent. This study was approved by the institutional review boards and ethics committees of the various centers. All patients had NC confirmed by either ultrasound, X-ray or CT, and all showed characteristic teeth findings with AI and delayed or missing tooth eruption.

In short, multipoint parametric linkage analysis utilizing 2,000 highly polymorphic markers (DeCode, Iceland) across the whole genome in 4 informative families as well as homozygosity mapping for another consanguineous family was used to determine the locus linked to this trait, as published before [4,5]. Next-generation sequencing using exome capture (Perkin Elmer, USA; Leeds Translational Genomics Unit, UK) was performed on 5 patients from 5 unrelated families; 4 were part of the linkage analysis. Subsequent data analysis was restricted to novel sequence variants within the linked region. The frequency of each variant was examined in >100 ethnically matched alleles available in public databases (1000 Genomes, release 12 – May 2012). Sanger sequencing was performed as described, and mutations were sequenced in family members for segregation analysis.

In detail, genomic DNA was isolated from peripheral blood lymphocytes for all subjects using standard protocols. Genotypes from polymorphic markers for 4 families were generated by DeCode, Iceland. Analyses were carried out as published before with modifications [4]. In short, genotypes were examined using a multipoint parametric linkage analysis and haplotype reconstruction was performed via Allegro and Genehunter for an autosomal recessive model with complete penetrance, disease allele frequency of 0.001 (DeCode map with appropriate allele frequencies). The data were formatted using Mega2 (version 4.0) through Alohomora (version 0.30, Win32); non-informative markers were filtered out. Mendelian inconsistencies were checked using PedCheck (version 1.1); unlikely genotypes were identified and filtered using Merlin (version 1.1 alpha 3). The Allegro haplotype output files were visualized with Haplopainter. In addition and in parallel, whole-genome SNP microarray analysis was performed on genomic DNA for another consanguineous family by AROS Applied Technology; resulting data were analyzed using IBD finder software [5]. Whole-exome sequencing was performed using 3 μg of genomic DNA, which was sheared and ligated to Illumina adapters, according to Agilent's SureSelect Library Prep protocol. The sample was then size selected (200-300 bp) by agarose gel electrophoresis and enriched for 12 cycles, using PCR prior to hybridization to the SureSelect reagent for 24 h at 65°C. The library was denatured using NaOH and diluted to a concentration of 12 pM, of which 120 μl was hybridized onto a v5 single-read flow cell (Illumina, San Diego, Calif., USA). Samples were prepared for sequencing according to Illumina's standard amplification, linearization, blocking and primer hybridization protocols. The flow cell was then loaded onto an Illumina GAIIx and sequencing performed for 80 cycles after which the raw data were processed using the Illumina pipeline. The data (qseq) files generated were aligned to the human reference sequence (hg19/GRCh37) using Novoalign short-read alignment software (Novocraft Technologies, Selangor, Malaysia). Duplicate reads and reads mapping to multiple locations were excluded from any analysis. SAMtools and the Genome Analysis Toolkit were used to further process the alignment files for variant calling [6,7].

For confirmation of variants detected by exome capture/next-generation sequencing and for mutation detection in additional cases, we amplified all coding exons and exon-intron boundaries of *FAM20A* using standard PCR methodology with intronic (genomic) primers. PCR products were separated on 1% agarose gels with ethidium bromide using electrophoresis and visualized under UV light. Specific bands were cut and DNA was isolated and purified using standard procedures. Bi-directional sequencing of all exons and exon-intron boundaries were performed using a Beckman Coulter CEQ8000 or an Applied Biosystems 3130xl capillary sequencer per the manufacturer's protocol. Sequencing data was analyzed and compared with the published reference sequence for *FAM20A* (NG_029809, April 2012, NCBI build 37.3).

## Results

We ascertained 25 patients (12 males, 13 females; age 12-64) in 16 families with NC and characteristic dental findings, i.e., the triad of AI, gingival thickening and impairment of tooth eruption (table 1). The diagnosis of NC was made predominantly by nephrologists based upon characteristic imaging findings. Generalized hypoplastic AI was evident from eruption of the deciduous teeth early in childhood with subsequent impaired eruption of the permanent teeth and development of gingival enlargement. Clinical details of some patients have been published [8,9,10,11]. None of the parents or offspring of our patients had AI.

**Fig. 1.**
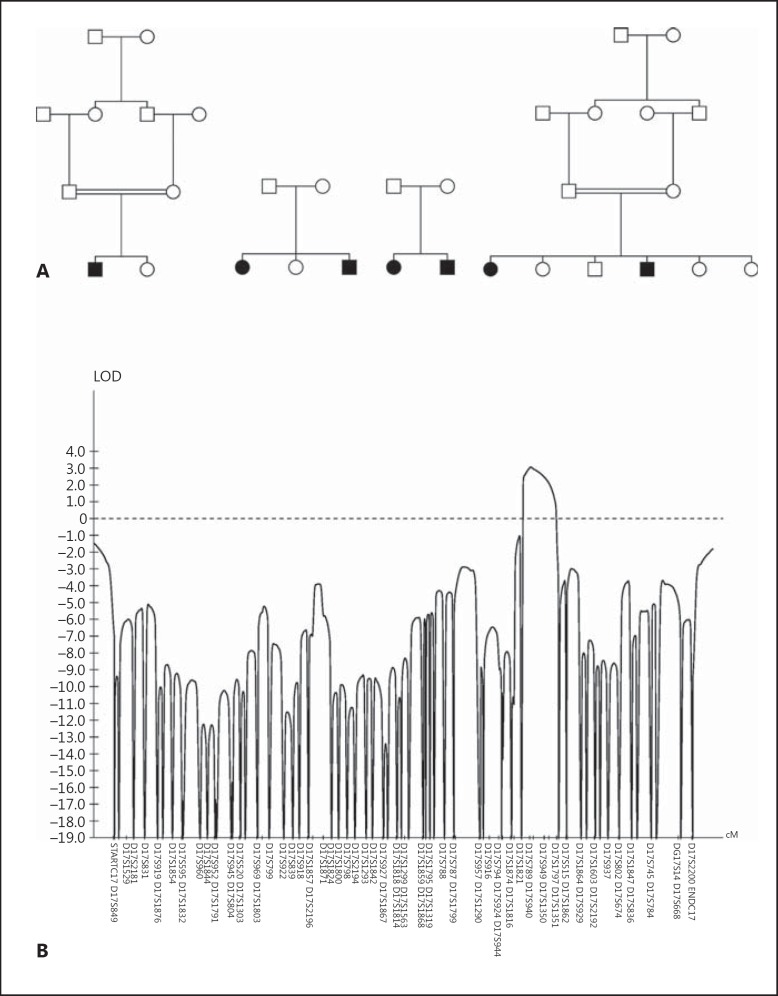
**A** Pedigrees used for multipoint parametric linkage analysis. Black symbols indicate affected, white unaffected, squares males and circles females. **B** LOD score analysis for chromosome 17. Note the single significant peak at 17q24.

From the 16 families with this disorder, we selected four informative families (fig. 1A) for whole-genome parametric multipoint linkage studies. Using dense (2 cM) polymorphic markers, we identified a single linked locus on chromosome 17q24 with a LOD score of 3.1 (fig. 1B). Haplotype reconstruction placed the locus between flanking markers D17S1821 (97.3 cM) and D17S1797 (106.2 cM), a region of 5.3 million bases containing 41 annotated genes (NCBI build 37.3, October 2011). Independently, homozygosity mapping identified the same locus in another consanguineous family (data not shown).

To identify the disease-causing gene, we performed massive parallel sequencing using exome capture on 5 unrelated patients. Using an autosomal recessive model, limiting our analysis to the linked region, and filtering variants to eliminate common polymorphisms, we found homozygous or compound heterozygous mutations in all 5 patients in only one gene, *FAM20A*. A total of 20 different mutations (deletions, insertions, and splice site, missense and nonsense mutations) were identified in the homozygous or compound heterozygous state, confirmed by Sanger sequencing, and demonstrated to -segregate with the disorder in our 16 families (table 1). *FAM20A* (cDNA 1,626 bp, protein 541 amino acids, 11 coding exons) is a member of a family of kinase-encoding genes that includes *FAM20B* and *FAM20C*.

## Discussion

Calcium plays many critical roles in human physiology, serving as an intracellular messenger, an extracellular neuromuscular excitatory ion, and a structural component of bone and teeth. For example, tooth enamel (calcium hydroxyapatite) is the hardest human tissue and can function into old age, despite being the only mineralized tissue with no capacity for cellular repair. Hence, the temporo-spatial concentration of calcium must be exquisitely regulated in different compartments, bound to albumin within the circulation, sequestered by calbindin within cells, including teeth [12], and excreted under tight control by the kidney [13]. Within the vasculature, calcium availability is closely modulated by an intricate interplay between bones and regulatory hormones that involves positive and negative feedback mechanisms (e.g., vitamin D, PTH, calcitonin) [14,15]. Consequences of dysregulated calcium homeostasis include nephrolithiasis and NC, i.e., precipitation of calcium in the urinary collecting system and renal interstitium, respectively.

The processes that normally maintain calcium balance in renal tissues can be delineated by studying genetic disorders involving NC. A prime example lies in an autosomal recessive disorder of NC combined with AI. Linkage analysis and focused exome sequencing performed on affected families identified the causative gene as *FAM20A*, previously associated only with a disorder of AI [16,17].

Previous causes of NC have involved epithelial and paracellular disturbances in calcium transport, predominantly caused by mutations in calcium-specific channels and proteins [18,19,20]. These systems either reabsorb filtered calcium from the filtrate (urine) across the renal tubular cell or release calcium from the tubular cell into the interstitial compartment. When they malfunction, increased urinary calcium precipitates within the renal tubule (leading to nephrolithiasis and urolithiasis) or within the interstitium (leading to NC); this is invariably accompanied by hypercalciuria.

Our 25 patients exhibited NC and AI, but ascertainment of other patients with biallelic *FAM20A* mutations will expand the phenotype and define the entire spectrum of this rare disease. Also, two different *Fam20a* knock-out mouse models exhibited different findings; in one, no kidney findings were reported, whereas the other showed arterial calcification without NC [21,22]. The patients previously reported with *FAM20A* mutations and AI may also prove to have NC.

## Conclusions

Our findings have implications for diagnosis and treatment. FAM20A is a locally secreted protein with low abundance in saliva [23] and blood [24], suggesting that replacement therapy could provide an option for a treatment or prophylaxis.

## Figures and Tables

**Table 1:** FAM20A mutations in patients with NC and AI

Family	Age, years	Gender	*FAM20A* mutations
1	21	male	c.915-918delCTTT; p.F305fsX380

2	27	female	IVS2 + 1G>A/c.913–914delTT; p.F305fsX378
	31	male	IVS2 + 1G>A/c.913–914delTT; p.F305fsX378

3	23	male	IVS4 + 1G>C/c.1348–1349delTC; p.S450fsX469
	25	female	IVS4 + 1G>C/c.1348–1349delTC; p.S450fsX469

4	59	male	c.1475–1482dupAACCCCAC; p.L495fsX509
	64	female	c.1475–1482dupAACCCCAC; p.L495fsX509

5	12	female	c.406C>T; p.R136X

6	20	male	c.34–35delCT; p.L12fsX78

7	16	female	c.1513delA; p.I505fsX506
	22	male	c.1513delA; p.I505fsX506

8	20	male	c.1432C>T; p.R478X

9	13	male	c.518T>G; p.L173R

10	29	female	c.727C>T/c.1228–-1229delGA; p.R243X/p.D410fsX414

11	19	female	c.217C>T/c.727C>T; p.R73X/p.R243X
	20	male	c.217C>T/c.727C>T; p.R73X/p.R243X

12	18	female	c.1369A>T; p.K457X

13	14	female	c.755–757delTCT/c.641-719del79bp; p.F252del/p.I214fsX259
	16	male	c.755–757delTCT/c.641–719del79bp; p.F252del/p.I214fsX259

14	21	female	IVS5 + 2T>G

15	24	male	c.907–908delAG; p.S303fsX378
	31	male	c.907–908delAG; p.S303fsX378
	37	female	c.907–908delAG; p.S303fsX378

16	17	female	c.34–35delCT/c.612delC; p.L12fsX78/p.A204fsX215
	18	female	c.34–35delCT/c.612delC; p.L12fsX78/p.A204fsX215

Mutations are described on the cDNA and predicted protein levels. Listing of one allele indicates homozygosity; two alleles indicate compound heterozygosity. Every patient had biallelic mutations involving insertions, deletions, essential splice sites, missense changes or nonsense changes.
